# Assessment of prioritizing the effective factors on human resources effectiveness (Case study: Tehran Industrial Parks Organization)

**DOI:** 10.1016/j.dib.2018.07.017

**Published:** 2018-07-21

**Authors:** Mehdi Pariav, Ahad Khalaji, S.Z. Hashemi, Majid Radfard

**Affiliations:** a Faculty of Management, Central Branch, Islamic Azad University, Tehran, Iran; b University of Social Welfare and Rehabilitation, Tehran, Iran; c School of public Health, Tehran University of Medical Science, Tehran, Iran

**Keywords:** Human Resources Management, Hay Group®, Effectiveness, Engagement, Enablement

## Abstract

Identifying the effective factors on human resources effectiveness can help management and leadership to obtain success, organization goals and fulfillment of high effectiveness and efficiency. Thus, they always have to survey the effective factors on effectiveness of these valuable and transformational resources. Effective factors on employee effectiveness have different aspects and varieties. For instance, Hay Group® model which is in order to compare organizations based on employee effectiveness. The model includes different factors located in two groups of ENGAGEMENT and ENABLEMENT. The main purpose of this study is to assess and prioritize effective factors on employee effectiveness in Tehran Industrial Parks. Furthermore, it is required to be surveyed and determined according to organizational properties and content dimensions of under study organization, and use of latent knowledge amongst organization experts (senior managers). This cross-sectional and descriptive- analytical research was performed in 2017. So, it is trying to achieve the purposes of study through interview, Delphi method, Multiple Criteria Decision-Making (MCDM) and Analytical Hierarchy Process (AHP).

**Specifications Table**

**Table:** 

Subject area	Human Resources Management
More specific subject area	Hay Group® model of Effectiveness, Iran Small Industries and Industrial Parks, Tehran Industrial Parks
Type of data	Tables, Diagram
How data was acquired	This cross-sectional and descriptive-analytical research was performed in 2017.
Data format	Raw, Analyzed
Data source location	Tehran Industrial Parks Organization as the main organization of Iran Small Industries and Industrial Parks (isipo) included 18 active industrial parks.
Data accessibility	Data is included in this article

**Value of the data**

• Investigating the factors of employee effectiveness in Iran Small Industries and Industrial Parks for the first time.
• Studying the global models of employee effectiveness and choose Hay Group® model as the basis.
• Using of Delphi and AHP techniques as selected research method in order to make effective decisions in Human Resources.
• Impact of effectiveness improvement in growth of organization and employee productivity.

## 1 Data

First, some demographic information about experts, including their position, department, work experience and degree are shown below in the following (Table 1, Table 2, Table 3, Table 4).

**Table 1:** Total numbers of participants according to position.

**Amount**	**Experts position**
1	CEO
2	Consultant
3	Assistant
12	Manager
**18**	**Total**

**Table 2:** Available participants according to departments.

**Amount**	**Experts department**
5	CEO zone
3	Deputy of Planning and Economic Affairs
4	Deputy of Small Industries
3	Deputy of Civil and Environmental
2	Deputy of Support and Human Resources
**17**	**Total**

**Table 3:** Participants work experience.

**Amount**	**Experts work experience (Year)**
4	8–12
4	13–17
6	18–23
3	Over 23
**17**	**Total**

**Table 4:** Participants degree.

**Amount**	**Experts degree**
5	B.Sc.
12	M.Sc.
**17**	**Total**

**Table 5:** Participants׳ responses to Delphi questionnaire.

**Table 6:** Raw data for paired comparisons matrix of criteria.

**Participants criteria**	**Likert scale**																	**Participants criteria**
Comprehensiveness	9	8	7	6	5	4	3	2	1	2	3	4	5	6	7	8	9	Influence level
Accessibility	9	8	7	6	5	4	3	2	1	2	3	4	5	6	7	8	9	Influence level
Accessibility	9	8	7	6	5	4	3	2	1	2	3	4	5	6	7	8	9	Comprehensiveness

**Table -7:** Participants’ responses for paired comparisons of criteria.

**Table 8:** Paired comparisons matrix of criteria.

**The participants criteria for effectiveness**	Influence level	Comprehensiveness	Accessibility	Mean	Percentage (%)
Influence level	1.00	1.14	0.74	0.96	31.59
Comprehensiveness	0.88	1.00	1.07	0.98	32.35
Accessibility	1.35	0.93	1.00	1.10	36.06

**Table 9:** Raw data for paired comparisons matrix of effective factors on human resources effectiveness (based on Hay Group effectiveness factors).

• **The criterion of "Influence level of employee effectiveness factors"**																		

1. Paired comparisons matrix between factors of employee engagement based on criterion of "Influence level"																		

Confidence in leaders	9	8	7	6	5	4	3	2	1	2	3	4	5	6	7	8	9	Clear and promising direction
Quality and customer focus	9	8	7	6	5	4	3	2	1	2	3	4	5	6	7	8	9	Clear and promising direction
Respect and recognition	9	8	7	6	5	4	3	2	1	2	3	4	5	6	7	8	9	Clear and promising direction
Development opportunities	9	8	7	6	5	4	3	2	1	2	3	4	5	6	7	8	9	Clear and promising direction
Pay and benefits	9	8	7	6	5	4	3	2	1	2	3	4	5	6	7	8	9	Clear and promising direction
Quality and customer focus	9	8	7	6	5	4	3	2	1	2	3	4	5	6	7	8	9	Confidence in leaders
Respect and recognition	9	8	7	6	5	4	3	2	1	2	3	4	5	6	7	8	9	Confidence in leaders
Development opportunities	9	8	7	6	5	4	3	2	1	2	3	4	5	6	7	8	9	Confidence in leaders
Pay and benefits	9	8	7	6	5	4	3	2	1	2	3	4	5	6	7	8	9	Confidence in leaders
Respect and recognition	9	8	7	6	5	4	3	2	1	2	3	4	5	6	7	8	9	Quality and customer focus
Development opportunities	9	8	7	6	5	4	3	2	1	2	3	4	5	6	7	8	9	Quality and customer focus
Pay and benefits	9	8	7	6	5	4	3	2	1	2	3	4	5	6	7	8	9	Quality and customer focus
Development opportunities	9	8	7	6	5	4	3	2	1	2	3	4	5	6	7	8	9	Respect and recognition
Pay and benefits	9	8	7	6	5	4	3	2	1	2	3	4	5	6	7	8	9	Respect and recognition
Pay and benefits	9	8	7	6	5	4	3	2	1	2	3	4	5	6	7	8	9	Development opportunities

2. Paired comparisons matrix between factors of employee enablement based on criterion of "Influence level"																		

Authority and empowerment	9	8	7	6	5	4	3	2	1	2	3	4	5	6	7	8	9	Performance management
Resources	9	8	7	6	5	4	3	2	1	2	3	4	5	6	7	8	9	Performance management
Training	9	8	7	6	5	4	3	2	1	2	3	4	5	6	7	8	9	Performance management
Collaboration	9	8	7	6	5	4	3	2	1	2	3	4	5	6	7	8	9	Performance management
Work, structure and processes	9	8	7	6	5	4	3	2	1	2	3	4	5	6	7	8	9	Performance management
Resources	9	8	7	6	5	4	3	2	1	2	3	4	5	6	7	8	9	Authority and empowerment
Training	9	8	7	6	5	4	3	2	1	2	3	4	5	6	7	8	9	Authority and empowerment
Collaboration	9	8	7	6	5	4	3	2	1	2	3	4	5	6	7	8	9	Authority and empowerment
Work, structure and processes	9	8	7	6	5	4	3	2	1	2	3	4	5	6	7	8	9	Authority and empowerment
Training	9	8	7	6	5	4	3	2	1	2	3	4	5	6	7	8	9	Resources
Collaboration	9	8	7	6	5	4	3	2	1	2	3	4	5	6	7	8	9	Resources
Work, structure and processes	9	8	7	6	5	4	3	2	1	2	3	4	5	6	7	8	9	Resources
Collaboration	9	8	7	6	5	4	3	2	1	2	3	4	5	6	7	8	9	Training
Work, structure and processes	9	8	7	6	5	4	3	2	1	2	3	4	5	6	7	8	9	Training
Work, structure and processes	9	8	7	6	5	4	3	2	1	2	3	4	5	6	7	8	9	Collaboration

• **The criterion of "Comprehensiveness of employee effectiveness factors"**																		

1. Paired comparisons matrix between factors of employee engagement based on criterion of "Comprehensiveness"																		

Confidence in leaders	9	8	7	6	5	4	3	2	1	2	3	4	5	6	7	8	9	Clear and promising direction
Quality and customer focus	9	8	7	6	5	4	3	2	1	2	3	4	5	6	7	8	9	Clear and promising direction
Respect and recognition	9	8	7	6	5	4	3	2	1	2	3	4	5	6	7	8	9	Clear and promising direction
Development opportunities	9	8	7	6	5	4	3	2	1	2	3	4	5	6	7	8	9	Clear and promising direction
Pay and benefits	9	8	7	6	5	4	3	2	1	2	3	4	5	6	7	8	9	Clear and promising direction
Quality and customer focus	9	8	7	6	5	4	3	2	1	2	3	4	5	6	7	8	9	Confidence in leaders
Respect and recognition	9	8	7	6	5	4	3	2	1	2	3	4	5	6	7	8	9	Confidence in leaders
Development opportunities	9	8	7	6	5	4	3	2	1	2	3	4	5	6	7	8	9	Confidence in leaders
Pay and benefits	9	8	7	6	5	4	3	2	1	2	3	4	5	6	7	8	9	Confidence in leaders
Respect and recognition	9	8	7	6	5	4	3	2	1	2	3	4	5	6	7	8	9	Quality and customer focus
Development opportunities	9	8	7	6	5	4	3	2	1	2	3	4	5	6	7	8	9	Quality and customer focus
Pay and benefits	9	8	7	6	5	4	3	2	1	2	3	4	5	6	7	8	9	Quality and customer focus
Development opportunities	9	8	7	6	5	4	3	2	1	2	3	4	5	6	7	8	9	Respect and recognition
Pay and benefits	9	8	7	6	5	4	3	2	1	2	3	4	5	6	7	8	9	Respect and recognition
Pay and benefits	9	8	7	6	5	4	3	2	1	2	3	4	5	6	7	8	9	Development opportunities

2. Paired comparisons matrix between factors of employee enablement based on criterion of "Comprehensiveness"																		

Authority and empowerment	9	8	7	6	5	4	3	2	1	2	3	4	5	6	7	8	9	Performance management
Resources	9	8	7	6	5	4	3	2	1	2	3	4	5	6	7	8	9	Performance management
Training	9	8	7	6	5	4	3	2	1	2	3	4	5	6	7	8	9	Performance management
Collaboration	9	8	7	6	5	4	3	2	1	2	3	4	5	6	7	8	9	Performance management
Work, structure and processes	9	8	7	6	5	4	3	2	1	2	3	4	5	6	7	8	9	Performance management
Resources	9	8	7	6	5	4	3	2	1	2	3	4	5	6	7	8	9	Authority and empowerment
Training	9	8	7	6	5	4	3	2	1	2	3	4	5	6	7	8	9	Authority and empowerment
Collaboration	9	8	7	6	5	4	3	2	1	2	3	4	5	6	7	8	9	Authority and empowerment
Work, structure and processes	9	8	7	6	5	4	3	2	1	2	3	4	5	6	7	8	9	Authority and empowerment
Training	9	8	7	6	5	4	3	2	1	2	3	4	5	6	7	8	9	Resources
Collaboration	9	8	7	6	5	4	3	2	1	2	3	4	5	6	7	8	9	Resources
Work, structure and processes	9	8	7	6	5	4	3	2	1	2	3	4	5	6	7	8	9	Resources
Collaboration	9	8	7	6	5	4	3	2	1	2	3	4	5	6	7	8	9	Training
Work, structure and processes	9	8	7	6	5	4	3	2	1	2	3	4	5	6	7	8	9	Training
Work, structure and processes	9	8	7	6	5	4	3	2	1	2	3	4	5	6	7	8	9	Collaboration

**•** **The criterion of "Accessibility to employee effectiveness factors"**																		

1. Paired comparisons matrix between factors of employee engagement based on criterion of "Accessibility"																		

Confidence in leaders	9	8	7	6	5	4	3	2	1	2	3	4	5	6	7	8	9	Clear and promising direction
Quality and customer focus	9	8	7	6	5	4	3	2	1	2	3	4	5	6	7	8	9	Clear and promising direction
Respect and recognition	9	8	7	6	5	4	3	2	1	2	3	4	5	6	7	8	9	Clear and promising direction
Development opportunities	9	8	7	6	5	4	3	2	1	2	3	4	5	6	7	8	9	Clear and promising direction
Pay and benefits	9	8	7	6	5	4	3	2	1	2	3	4	5	6	7	8	9	Clear and promising direction
Quality and customer focus	9	8	7	6	5	4	3	2	1	2	3	4	5	6	7	8	9	Confidence in leaders
Respect and recognition	9	8	7	6	5	4	3	2	1	2	3	4	5	6	7	8	9	Confidence in leaders
Development opportunities	9	8	7	6	5	4	3	2	1	2	3	4	5	6	7	8	9	Confidence in leaders
Pay and benefits	9	8	7	6	5	4	3	2	1	2	3	4	5	6	7	8	9	Confidence in leaders
Respect and recognition	9	8	7	6	5	4	3	2	1	2	3	4	5	6	7	8	9	Quality and customer focus
Development opportunities	9	8	7	6	5	4	3	2	1	2	3	4	5	6	7	8	9	Quality and customer focus
Pay and benefits	9	8	7	6	5	4	3	2	1	2	3	4	5	6	7	8	9	Quality and customer focus
Development opportunities	9	8	7	6	5	4	3	2	1	2	3	4	5	6	7	8	9	Respect and recognition
Pay and benefits	9	8	7	6	5	4	3	2	1	2	3	4	5	6	7	8	9	Respect and recognition
Pay and benefits	9	8	7	6	5	4	3	2	1	2	3	4	5	6	7	8	9	Development opportunities

2. Paired comparisons matrix between factors of employee enablement based on criterion of "Accessibility"																		

Authority and empowerment	9	8	7	6	5	4	3	2	1	2	3	4	5	6	7	8	9	Performance management
Resources	9	8	7	6	5	4	3	2	1	2	3	4	5	6	7	8	9	Performance management
Training	9	8	7	6	5	4	3	2	1	2	3	4	5	6	7	8	9	Performance management
Collaboration	9	8	7	6	5	4	3	2	1	2	3	4	5	6	7	8	9	Performance management
Work, structure and processes	9	8	7	6	5	4	3	2	1	2	3	4	5	6	7	8	9	Performance management
Resources	9	8	7	6	5	4	3	2	1	2	3	4	5	6	7	8	9	Authority and empowerment
Training	9	8	7	6	5	4	3	2	1	2	3	4	5	6	7	8	9	Authority and empowerment
Collaboration	9	8	7	6	5	4	3	2	1	2	3	4	5	6	7	8	9	Authority and empowerment
Work, structure and processes	9	8	7	6	5	4	3	2	1	2	3	4	5	6	7	8	9	Authority and empowerment
Training	9	8	7	6	5	4	3	2	1	2	3	4	5	6	7	8	9	Resources
Collaboration	9	8	7	6	5	4	3	2	1	2	3	4	5	6	7	8	9	Resources
Work, structure and processes	9	8	7	6	5	4	3	2	1	2	3	4	5	6	7	8	9	Resources
Collaboration	9	8	7	6	5	4	3	2	1	2	3	4	5	6	7	8	9	Training
Work, structure and processes	9	8	7	6	5	4	3	2	1	2	3	4	5	6	7	8	9	Training
Work, structure and processes	9	8	7	6	5	4	3	2	1	2	3	4	5	6	7	8	9	Collaboration

**Table 16:** Priority of engagement factors of employee effectiveness based on prioritized criteria.

**Factors of employee engagement**							
**The participants criteria for effectiveness**	Priority of criteria	Clear and promising direction	Confidence in leaders	Quality and customer focus	Respect and recognition	Development opportunities	Pay and benefits
Accessibility	1	12.94	11.81	13.03	17.06	20.31	24.85
	Priority of factors	5	6	4	3	2	1
Comprehensiveness	2	10.69	14.85	9.13	18.88	18.69	27.76
	Priority of factors	5	4	6	2	3	1
Influence level	3	9.72	14.37	5.47	18.24	21.95	30.25
	Priority of factors	5	4	6	3	2	1

**Table 17:** Priority of enablement factors of employee effectiveness based on prioritized criteria.

**Factors of employee enablement**							
**The participants criteria for effectiveness**	Priority of criteria	Performance management	Authority and empowerment	Resources	Training	Collaboration	Work, structure and processes
Accessibility	1	15.13	14.61	21.21	21.56	14.08	13.41
	Priority of factors	3	4	2	1	5	6
Comprehensiveness	2	12.75	17.88	13.37	28.35	15.31	12.34
	Priority of factors	5	2	4	1	3	6
Influence level	3	11.11	13.00	12.37	27.28	19.94	16.30
	Priority of factors	6	4	5	1	2	3

Table 1 shows total numbers of senior managers and experts of research community according to their position.

Table 2 indicates available experts based on their departmentsand due to interview time and willingness of participating in the study.

Table 3 expresses experts work experience within management positions for years.

Also, Table 4 describes experts׳ last degree; though some of these experts were studying PhD in their own expertise.

In Delphi process and after initial interviews, experts have reached a consensus and declared three criteria (choices) accepted to be scored and compared for ranking. Table 5 shows participants responses to Delphi questionnaire.

**AHP tables:** (Table 6, Table -7, Table 8, Table 9)

In AHP process, accepted criteria were compared in pair, and their rank was extracted. Then, comparison of factors (alternatives) has done according to each criterion. So, alternatives or factors were ranked separately.

After consensus, participants (experts) in interview have stated common criteria for identifying factors affecting on employee effectiveness. Prioritized criteria in this research collected by experts based on Delphi method are included:

• Accessibility
• Comprehensiveness
• Influence level

According to Table 10, the priority of the factors affecting on employee engagement based on criterion of "Accessibility" are respectively:

1. Pay and benefits
2. Development opportunities
3. Respect and recognition
4. Quality and customer focus
5. Clear and promising direction
6. Confidence in leaders

**Table 10:** The result of paired comparisons matrix between factors of employee engagement based on criterion of "Accessibility".

1. The criterion of "Accessibility to factors of employee effectiveness"									
**Factors of employee engagement**	Clear and promising direction	Confidence in leaders	Quality and customer focus	Respect and recognition	Development opportunities	Pay and benefits	Geometric mean	Percentage (%)	Rank
Clear and promising direction	1.00	0.54	1.77	0.40	0.50	0.39	0.99	12.94	5
Quality and customer focus	0.57	0.57	1.00	0.55	0.58	0.29	1.00	13.03	4
Respect and recognition	2.50	0.87	1.81	1.00	1.35	0.87	1.30	17.06	3
Development opportunities	2.01	1.19	1.72	0.74	1.00	0.57	1.55	20.31	2
Pay and benefits	2.56	1.50	3.41	1.15	1.77	1.00	1.90	24.85	1

According to Table 11, the priority of the factors affecting on employee enablement based on criterion of "Accessibility" are respectively:

1. Training
2. Resources
3. Performance management
4. Authority and empowerment
5. Collaboration
6. Work, structure and processes

**Table 11:** The result of paired comparisons matrix between factors of employee enablement based on criterion of "Accessibility".

2. The criterion of "Accessibility to factors of employee effectiveness"									
**Factors of employee enablement**	Performance management	Authority and empowerment	Resources	Training	Collaboration	Work, structure and processes	Geometric mean	Percentage (%)	Rank
Performance management	1	1.23	1.20	0.40	0.93	1.10	0.98	15.13	3
Authority and empowerment	0.81	1	0.97	0.98	1.01	1.14	0.95	14.61	4
Resources	0.83	1.03	1	0.78	1.04	0.77	1.37	21.21	2
Training	2.48	1.02	1.28	1	2.53	2.71	1.4	21.56	1
Collaboration	1.08	0.99	0.96	0.40	1	1.31	0.91	14.08	5
Work, structure and processes	0.91	0.88	1.29	0.37	0.77	1	0.87	13.41	6

According to Table 12, the priority of the factors affecting on employee engagement based on criterion of "Comprehensiveness" are respectively:

1. Pay and benefits
2. Respect and recognition
3. Development opportunities
4. Confidence in leaders
5. Clear and promising direction
6. Quality and customer focus

**Table 12:** The result of paired comparisons matrix between factors of employee engagement based on criterion of "Comprehensiveness".

1. The criterion of "Comprehensiveness of employee effectiveness factors"									
**Factors of employee engagement**	Clear and promising direction	Confidence in leaders	Quality and customer focus	Respect and recognition	Development opportunities	Pay and benefits	Geometric mean	Percentage (%)	Rank
Clear and promising direction	1	0.92	1.46	0.35	0.53	0.30	0.76	10.69	5
Quality and customer focus	0.68	0.45	1	0.79	0.57	0.39	0.65	9.13	6
Respect and recognition	2.89	1.21	1.27	1	1.13	0.55	1.34	18.88	2
Development opportunities	1.90	1.83	1.74	0.89	1	0.60	1.33	18.69	3
Pay and benefits	3.28	1.51	2.56	1.80	1.66	1	1.97	27.76	1

According to Table 13, the priority of the factors affecting on employee enablement based on criterion of "Comprehensiveness" are respectively:

1. Training
2. Authority and empowerment
3. Collaboration
4. Resources
5. Performance management
6. Work, structure and processes

**Table 13:** The result of paired comparisons matrix between factors of employee enablement based on criterion of "Comprehensiveness".

2. The criterion of "Comprehensiveness of employee effectiveness factors"									
**Factors of employee enablement**	Performance management	Authority and empowerment	Resources	Training	Collaboration	Work, structure and processes	Geometric mean	Percentage (%)	Rank
Performance management	1	0.64	1.38	0.44	0.89	0.73	0.85	12.75	5
Authority and empowerment	1.56	1	1.41	0.68	1.03	1.46	1.19	17.88	2
Resources	0.73	0.71	1	0.69	0.92	1.29	0.89	13.37	4
Training	2.25	1.46	1.45	1	3.05	2.09	1.88	28.35	1
Collaboration	1.12	0.97	1.09	0.33	1	1.60	1.02	15.31	3
Work, structure and processes	1.36	0.69	0.77	0.48	0.62	1	0.82	12.34	6

According to Table 14, the priority of the factors affecting on employee engagement based on criterion of "Influence level" are respectively:

1. Pay and benefits
2. Development opportunities
3. Respect and recognition
4. Confidence in leaders
5. Clear and promising direction
6. Quality and customer focus

**Table 14:** The result of paired comparisons matrix between factors of employee engagement based on criterion of "Influence level".

1. The criterion of "Influence level of employee effectiveness factors"									
**Factors of employee engagement**	Clear and promising direction	Confidence in leaders	Quality and customer focus	Respect and recognition	Development opportunities	Pay and benefits	Geometric mean	Percentage (%)	Rank
Clear and promising direction	1	0.76	1.93	0.45	0.37	0.28	0.80	9.72	5
Quality and customer focus	0.52	0.32	1	0.37	0.27	0.22	0.45	5.47	6
Respect and recognition	2.20	1.62	2.72	1	1.01	0.44	1.50	18.24	3
Development opportunities	2.68	1.80	3.75	0.99	1	0.61	1.80	21.95	2
Pay and benefits	3.54	2.00	4.46	2.27	1.64	1	2.49	30.25	1

According to Table 15, the priority of the factors affecting on employee enablement based on criterion of "Influence level" are respectively: (Table 16, Table 17)

1. Training
2. Collaboration
3. Work, structure and processes
4. Authority and empowerment
5. Resources
6. Performance management

**Table 15:** The result of paired comparisons matrix between factors of employee enablement based on criterion of "Influence level".

2. The criterion of "Influence level of employee effectiveness factors"									
**Factors of employee enablement**	Performance management	Authority and empowerment	Resources	Training	Collaboration	Work, structure and processes	Geometric mean	Percentage (%)	Rank
Performance management	1	0.80	0.78	0.31	0.67	0.90	0.74	11.11	6
Authority and empowerment	1.26	1	1.05	0.56	0.67	0.68	0.87	13.00	4
Resources	1.28	0.95	1	0.53	0.66	0.55	0.83	12.37	5
Training	3.24	1.79	1.88	1	1.26	1.77	1.82	27.28	1
Collaboration	1.48	1.49	1.52	0.79	1	1.72	1.33	19.94	2
Work, structure and processes	1.11	1.46	1.83	0.56	0.58	1	1.09	16.30	3

## 2 Materials and methods

This cross-sectional and descriptive-analytical research was performed at Tehran Industrial Parks Organization in 2017. This organization has 18 active scattered industrial parks in Tehran province which are heterogeneous in terms of some features. The study sampling method for determining prioritized criteria was purposive, non-random and non-probable. For this purpose, the statistical population has been selected among senior managers of the research community in the sample of 18 people whom were asked by designed questionnaire and interview through Delphi method and Hierarchy Analytical Process (AHP) to score their criteria and determine the priority and rank of each criterion(choice) and factor(alternative). Subsequently, by multivariate decision the weight of each criterion and weights has been obtained and the criteria are analyzed according to purpose of the effectiveness of human resources and the priority is determined accordingly [1], [2], [3], [4], [5], [6], [7], [8], [9], [10], [11], [12]
