# Supplementary material for: Assessment of prioritizing the effective factors on human resources effectiveness (Case study: Tehran Industrial Parks Organization)
Source: Data Brief. 2018 Jul 21;19:2455–67. doi: 10.1016/j.dib.2018.07.017 (PMC6141523; doi:10.1016/j.dib.2018.07.017)
Supplement: Supplementary file 1 — Supplementary material [file mmc1.docx]

**Conflict of Interest*:**

The authors of this article declare that they have no conflict of interests.

**Conflict of Interest*:**

Declarations of interest: none
